# Supplementary material for: Localization of spontaneous bursting neuronal activity in the preterm human brain with simultaneous EEG-fMRI
Source: eLife. 2017 Sep 12;6:e27814. doi: 10.7554/eLife.27814 (PMC5595428; doi:10.7554/eLife.27814)
Supplement: Supplementary file 1. [file elife-27814-supp1.docx]

| Number of infants | 13 |
| --- | --- |
| Median (range) GA at birth (weeks + days) | 31+2 (28+2 to 34+4) |
| Median (range) birth weight (g) | 1645 (1080 to 2110) |
| Number of female | 5 |
| Median (range) PMA at time of study (weeks + days) | 35 (32+2 to 36+2) |
| Median (range) postnatal age at time of study (days) | 18 (from 5 to 55) |

**Supplementary Table 1:** Demographic information of the study sample
