# Supplementary material for: Localization of spontaneous bursting neuronal activity in the preterm human brain with simultaneous EEG-fMRI
Source: eLife. 2017 Sep 12;6:e27814. doi: 10.7554/eLife.27814 (PMC5595428; doi:10.7554/eLife.27814)
Supplement: Supplementary file 2. — In bold are topographical distributions that occurred at least three times for a given subject and were therefore used in the fMRI first level (individual subject) analysis. Delta brushes were unilateral (right – R or left – L), midline (M) or bilateral (B) frontal (F), central (C), temporal (T), parietal (Pa), posterior-temporal (PT), occipital (O), posterior-temporal occipital (PTO) or involving the posterior quadrant (PQ). [file elife-27814-supp2.docx]

| **Subject ID #** | **PMA at time of study**  **(weeks+days)** | **Length of recording**  **(volumes / time)** | **Length of accepted recording**  **(volumes / time)** | **Delta Brush topography /**  **number** |
| --- | --- | --- | --- | --- |
| 1291 | 32+2 | 399 / 10 min | 288 / 7 min | **B-PT / 5**  **L-PT / 15**  **L-T / 5**  R-Pa / 2  **R-PT / 5** |
| 1076 | 33+1 | 299 / 7.5 min | 299 / 7.5 min | B-O / 2  B-PQ / 3  B-PTO / 3  L-C / 1  L-O / 1  L-Pa / 1  L-PQ / 1  **L-PT / 15**  L-PTO / 1  L-T / 1  M-Pa / 3  R-O / 3  R-PQ / 1  **R-PT / 9**  **R-PTO / 5** |
| 1170 | 34+6 | 299 / 7.5 min | 299 / 7.5 min | **B-O / 4**  B-PaO / 1  B-PQ / 1  B-PTO / 1  **L-O / 6**  **L-PT / 7**  L-PTO / 3  R-Pa / 1  R-PaO / 1  **R-PT / 13** |
| 0286 | 35+0 | 299 / 7.5 min | 138 / 3.5 min | **L-PT / 5**  **R-PT / 6** |
| 1288 | 35+0 | 499 / 12.5 min | 414 / 10.5 min | B-Pa / 1  B-PaO / 1  B-PTO / 1  **L-Pa / 4**  **L-PT / 8**  L-F / 2  L-T / 3  R-O / 1  R-Pa / 2  **R-PT / 4**  B-PQ / 3 |
| 1290 | 35+1 | 499 / 12.5 min | 199 / 5 min | B-PT / 3  **L-PT / 7**  **R-PT / 11** |
| 0402 | 35+2 | 299 / 7.5 min | 299 / 7.5 min | B-O / 1  L-O / 2  **L-PT / 4**  **R-PT / 5**  R-PTO / 2 |
| 1298 | 35+6 | 399 / 10 min | 399 / 10 min | **B-O / 9**  **B-PT / 12**  B-PTO / 1  L-O / 2  **L-PT / 10**  L-PTO / 2  R-O / 1  **R-PT / 15**  **R-PTO / 5** |
| 1053 | 36+1 | 299 / 7.5 min | 299 / 7.5 min | L-O / 1  **L-PQ / 7**  M-Pa / 1  R-CP / 2  **R-Pa / 5**  R-PQ / 2  **R-PT / 6** |
| 1307 | 36+2 | 399 / 10 min | 138 / 3.5 min | B-PT / 2  **L-PT / 7**  **R-PT / 12** |

**Supplementary Table 2**: **Delta brush topographical distributions**. In bold are topographical distributions that occurred at least three times for a given subject and were therefore used in the fMRI first level (individual subject) analysis. Delta brushes were unilateral (right – R or left – L), midline (M) or bilateral (B) frontal (F), central (C), temporal (T), parietal (Pa), posterior-temporal (PT), occipital (O), posterior-temporal occipital (PTO) or involving the posterior quadrant (PQ).
