# Supplementary material for: Localization of spontaneous bursting neuronal activity in the preterm human brain with simultaneous EEG-fMRI
Source: eLife. 2017 Sep 12;6:e27814. doi: 10.7554/eLife.27814 (PMC5595428; doi:10.7554/eLife.27814)
Supplement: Supplementary file 3. — Summary of the spatial location of significant clusters identified in the first level analysis of the EEG-fMRI data for each subject with respect to the explanatory variables included in the model (delta brush topography). [file elife-27814-supp3.docx]

| **Subject ID #** | **Delta brush EEG topography** | **FMRI results** |
| --- | --- | --- |
| 1291 | **L-PT** | L inferior frontal, L prefrontal |
|  | **R-PT** | R posterior insula, R posterior parietal, L frontal, SMA, precuneus |
|  | **B-PT** | Bi posterior parietal, posterior cingulate |
|  | **L-T** | -- |
| 1076 | **L-PT** | L posterior insula, L parietal operculum |
|  | **R-PT** | R anterior insula, R inferior temporal, L posterior insula, L superior temporal gyrus, L parietal, precuneous, anterior cingulate |
|  | **R-PTO** | -- |
| 1170 | **L-PT** | L posterior insula, L superior temporal gyrus, L cerebellum, R inferior temporal, R frontal, SMA |
|  | **R-PT** | R temporal pole, R anterior insula, R posterior temporal, R peri-rolandic, L anterior insula, L peri-rolandic, L occipital, SMA, precuneus |
|  | **B-O** | -- |
|  | **L-O** | -- |
| 0286 | **L-PT** | L temporal pole, L posterior insula, L basal ganglia |
|  | **R-PT** | R posterior temporal, R frontal, anterior cingulate, L parietal |
| 1288 | **L-PT** | R posterior insula, L thalamus |
|  | **R-PT** | R temporal pole, L temporal pole, medial prefrontal |
|  | **L-Pa** | R parietal |
| 1290 | **L-PT** | L inferior temporal |
|  | **R-PT** | R temporal pole, R superior temporal gyrus, R posterior insula, R frontal parietal, L parietal operculum, anterior cingulate |
| 0402 | **L-PT** | L temporal pole, L posterior insula, L inferior temporal, L posterior parietal, R temporal pole |
|  | **R-PT** | -- |
| 1298 | **L-PT** | Bi occipital |
|  | **R-PT** | R insula, R parietal operculum, L anterior insula, L occipital, L inferior frontal |
|  | **B-PT** | Bi insula, Bi superior temporal, Bi occipital, Bi parietal |
|  | **R-PTO** | -- |
|  | **B-O** | Medial occipital, Bi posterior parietal |
| 1053 | **R-PT** | R temporal pole, R frontal, R thalamus, L anterior insula |
|  | **R-Pa** | R parietal operculum, R temporal pole, R posterior temporal |
|  | **L-PQ** | L perirolandic, L anterior insula, L inferior temporal, R anterior insula, R inferior temporal, cingulate |
| 1307 | **L-PT** | L anterior insula, L parietal operculum, L inferior frontal, R anterior insula |
|  | **R-PT** | R posterior insula, L temporal pole, anterior cingulate |

**Supplementary Table 3: First level fMRI analysis results**. Summary of the spatial location of significant clusters identified in the first level analysis of the EEG-fMRI data for each subject with respect to the explanatory variables included in the model (delta brush topography).
