# Supplementary material for: Localization of spontaneous bursting neuronal activity in the preterm human brain with simultaneous EEG-fMRI
Source: eLife. 2017 Sep 12;6:e27814. doi: 10.7554/eLife.27814 (PMC5595428; doi:10.7554/eLife.27814)
Supplement: Transparent reporting form [file elife-27814-transrepform.pdf]

1st Floor
24 Hills Road
Cambridge CB2 1JP, UK

P 01223 855340
W elifesciences.org
T @elife

eLife's transparent reporting form

We encourage authors to provide detailed information *within their submission* to facilitate the interpretation and replication of experiments. If you have any questions, please contact us: editorial@elifesciences.org.

Sample-size estimation

- You should state whether an appropriate sample size was computed when the study was being designed
- You should state the statistical method of sample size computation and any required assumptions
- If no explicit power analysis was used, you should describe how you decided what sample (replicate) size (number) to use

Please outline where this information can be found within the submission (e.g., page numbers or figure legends), or explain why this information doesn't apply to your submission:

- As the study constituted entirely novel data in terms of the methods used and the population it was applied to, it was not possible to perform a true power calculation prior to data collection.
- We therefore estimated sample size using statistical maps derived from task-based fMRI data acquired in a previous study of preterm human infants (Allievi et al. Cerebral Cortex 2016). Using this data with an open-source fMRI power calculation tool (http://neuropowertools,org/), there is a predicted power of 0.8-0.9 for 10-12 patients (with a conservative cluster based screening threshold of z>2.3 and Random Field theory correction, p<0.05).

Replicates

- You should report how often each experiment was performed
- You should include a definition of biological versus technical replication
- The data obtained should be provided and sufficient information should be provided to indicate the number of independent biological and/or technical replicates
- If you encountered any outliers, you should describe how these were handled
- Criteria for exclusion/inclusion of data should be clearly stated
- High-throughput sequence data should be uploaded before submission, with a private link for reviewers provided (these are available from both GEO and ArrayExpress)

eLife Sciences Publications, Ltd is a limited liability non-profit non-stock corporation incorporated in the State of Delaware, USA, with company number 5030732, and is registered in the UK with company number FC030576 and branch number BR015634 at the address 1st Floor, 24 Hills Road, Cambridge CB2 1JP | August 2014

Please outline where this information can be found within the submission (e.g., page numbers or figure legends), or explain why this information doesn't apply to your submission:

- *Information about the data collection and replication has been detailed in the manuscript in both the main body and on-line methods/materials. Specific information and its' reference in the manuscript is detailed below.*
- Due to the fragile nature of our study population (preterm human infants which are at high risk of hypothermia, hypoglycaemia, and infection when removed from their normal clinical environment), data was collected during a single imaging session on a 3T Philips MR scanner located on the Neonatal Intensive Care Unit at St Thomas' Hospital London. During the imaging session, structural images for clinical interpretation (TSE (turbo spin echo) T2 weighted and MPRAGE (Magnetization Prepared Rapid Acquisition Gradient Echo) T1 weighted sequences) were acquired in addition to simultaneous EEG-fMRI data which was acquired using a GRE-EPI (Gradient Echo Echo-Planar-Imaging) sequence lasting up to 12.5 minutes (*on-line methods and materials page 15 of the manuscript*).
- Across our study group, the neural events of interest had an incidence of 4.4 per minute (range 1.9 to 6.7). Although independent technical replication was not possible due to the aforementioned clinical factors, independent biological replication was achieved through acquiring data using the same experimental set-up in a total of 13 infants (with successful data collection in 10) (*main body of the manuscript results section page 5*).
- Data was discarded in 3 cases: in 2 cases due to insufficient EEG quality (due to bridging electrodes or unsuccessful MR gradient artifact removal); and in 1 case due to an insufficient number of neural events following data pre-processing (which included removal of data corrupted by excessive head motion affecting both the EEG and fMRI recordings). No data was excluded for outlier reasons (*on-line methods and materials page 16 and page 18*).
- There was no high-throughput sequence data in this study.

Statistical reporting

- Statistical analysis methods should be described and justified
- Raw data should be presented in figures whenever informative to do so (typically when N per group is less than 10)
- For each experiment, you should identify the statistical tests used, exact values of N, definitions of center, methods of multiple test correction, and dispersion and precision measures (e.g., mean, median, SD, SEM, confidence intervals; and, for the major substantive results, a measure of effect size (e.g., Pearson's r, Cohen's d)

- Report exact p-values wherever possible alongside the summary statistics and 95% confidence intervals. These should be reported for all key questions and not only when the p-value is less than 0.05.

Please outline where this information can be found within the submission (e.g., page numbers or figure legends), or explain why this information doesn't apply to your submission:

- All fMRI data analysis was performed using tools within FSL (FMRIB's software library, www.fmrib.ox.ac.uk/fsl).
- Individual subject data was analyzed using the general linear model (GLM) as implemented in FEAT (v5.98) by defining an explanatory variable (model regressor) from the convolution of the identified neural events and a population specific HRF (Arichi et al. Neuroimage 2012). Standard cluster-based thresholding ($z>2.3$, corrected cluster significance level $p<0.005$) was used to identify significant areas of functional activity (*on-line methods/materials page 18*).
- The mean group effect was identified using a non-parametric one sample T-test as implemented in Randomise (v2.1), which utilizes permutation methods to provide inference on data where the null distribution is not known. Significant effects ($p<0.05$) were identified following family-wise error correction for multiple comparisons and using threshold free cluster enhancement (TFCE, Smith & Nichols Neuroimage 2009) (*on-line methods/materials page 21*).

(For large datasets, or papers with a very large number of statistical tests, you may upload a single table file with tests, Ns, etc., with reference to page numbers in the manuscript.)

Additional data files ("source data")

- We encourage you to upload relevant additional data files, such as numerical data that are represented as a graph in a figure, or as a summary table
- Where provided, these should be in the most useful format, and they can be uploaded as "Source data" files linked to a main figure or table
- Include model definition files including the full list of parameters used
- Include code used for data analysis (e.g., R, MatLab)
- Avoid stating that data files are "available upon request"

Please indicate the figures or tables for which source data files have been provided:

- A table containing the location and incidence of specific delta brush topographies for each subject have been included in the on-line methods and materials (*supplementary table 2*).
- A table detailing the location of significant clusters identified in the fMRI data for each individual subject has also been included in the on-line methods and materials (*supplementary table 3*).
- Imaging data will be publically available on an institutional repository (brain-development.org).
